# In This Issue

**Published:** 2001

## ALCOHOL AND HEPATITIS C

Infection with the hepatitis C virus (HCV) is a common cause of liver disease—including cirrhosis—and of cirrhosis-related death in the United States. As Dr. Charles S. Lieber reports, HCV infection is particularly common among alcoholics, and alcohol can exacerbate the consequences of HCV infection through several mechanisms. For example, the breakdown of alcohol in the liver produces toxic molecules that can lead to liver damage. It also can increase the damage associated with HCV infection by exacerbating the inflammation and formation of scar tissue associated with HCV infection, which, in turn, may accelerate the disease progression to cirrhosis and liver cancer. Chronic alcohol consumption also interferes with treatment for HCV by promoting liver disease, reducing the effectiveness of a drug commonly used to treat HCV infection (i.e., interferon-alpha), and by hampering the patient’s ability to adhere to a treatment regimen. (pp. 245–254)

## ALCOHOL’S EFFECTS ON THE RISK FOR CORONARY HEART DISEASE

A variety of studies, involving thousands of people, have shown that moderate drinkers have a lower risk of coronary heart disease than do people who abstain from drinking. Other studies have investigated whether it is alcohol consumption alone or other related factors (e.g., dietary and lifestyle factors) that reduce the risk of heart disease. Drs. Kenneth J. Mukamal and Eric B. Rimm analyzed existing studies on the effects of alcohol consumption on various biological markers that influence the risk of cardiovascular disease. Their analysis found that alcohol may increase the levels of “good cholesterol” and reduce other risk factors for heart disease. At the same time, alcohol may increase the levels of certain fat molecules, increasing the risk of disease. Overall, the findings indicated that alcohol indeed may lower the risk of heart disease in moderate drinkers. However, genetic factors (e.g., differences in the enzymes that break down alcohol in the body) also play an important role and may modify the positive effect that alcohol has on coronary heart disease. (pp. 255–261)

## ALCOHOL CONSUMPTION AND THE RISK OF CANCER

Heavy drinking has been associated with an increased risk for various disorders, including several types of cancer. Mr. Vincenzo Bagnardi, Ms. Marta Blangiardo, and Drs. Carlo La Vecchia and Giovanni Corrao review the findings from more than 200 studies that investigated the effects of various levels of alcohol consumption on the risk for cancer. Their investigation found that alcohol was linked most strongly with cancers of the oral cavity, pharynx, esophagus, and larynx. The risk for developing other cancers (e.g., stomach, colon, rectum, liver, breast, and ovaries) was lower. They found too that drinking approximately four drinks per day or more significantly increased the risk of developing any type of cancer. The authors note that alcohol alone does not appear to cause cancer, but may instead act as a co-carcinogen—a substance that promotes or accelerates cancer when administered together with other cancer-causing substances. (pp. 263–270)

## ALCOHOL AND FEMALE PUBERTY

Drinking during adolescence has far-reaching and detrimental health effects. In this article, Drs. W. Les Dees, Vinod K. Srivastava, and Jill K. Hiney review how drinking can delay puberty in girls by suppressing normal growth of the ovaries. Alcohol inhibits the production of hormones that regulate ovarian function. Those hormones are secreted by the pituitary gland and reach the ovaries through the bloodstream. According to the authors, alcohol also can sabotage important mechanisms located entirely within the ovary. Although alcohol influences each of these “intraovarian” systems differently, the combined effect of the changes results in pubertal delay and poorly coordinated development of the reproductive system. The long-term consequences of this phenomenon are not yet known. However, the discovery of this new mechanism helps confirm one of alcohol’s recognized adverse effects on young people, and underscores the need for effective outreach and education programs to better educate youth about the risks of drinking. (pp. 271–275)

## EFFECTS OF ALCOHOL USE AND ESTROGEN ON BONE

Studies show that women who drink alcohol may have less bone loss than women who abstain from drinking. The beneficial effects of alcohol use are especially apparent in postmenopausal women. Estrogen deficiency accompanying menopause leads to bone loss, which then predisposes women to developing osteoporosis later in life. Drs. Russell T. Turner and Jean D. Sibonga describe the process of bone remodeling, in which small areas of bone are destroyed and rebuilt, and they explain the potential mechanisms by which alcohol might reduce bone loss in post-menopausal women. The authors also review alcohol’s negative effects on bone, including how it may be harmful to growing bone and how it may have a negative effect on bone health in men. (pp. 276–281)

## ALCOHOL AND THE MALE REPRODUCTIVE SYSTEM

Alcohol use affects the three key players in the body’s reproductive system—the hypothalamus, the pituitary gland, and the gonads (e.g., the testes). Alcohol use is associated with low testosterone and altered levels of additional reproductive hormones. Drs. Mary Ann Emanuele and Nicholas Emanuele describe alcohol’s effects on the male reproductive system and review several potential mechanisms for alcohol’s damage. Those mechanisms are related to alcohol metabolism, alcohol-related cell damage, and other hormonal reactions associated with alcohol consumption. The authors also describe new strategies for preventing or reversing alcohol’s harmful effects on the reproductive system and review research in male rats on the effects of chronic alcohol use on reproductive health and on the health of their offspring. (pp. 282–287)

## EFFECTS OF ALCOHOL AND HIV INFECTION ON THE CENTRAL NERVOUS SYSTEM

Alcohol is known to have deleterious effects on the body’s immune system. Heavy alcohol use also may adversely affect mental functions and behavior. A common cause of illness and death associated with HIV infection is a syndrome characterized by impaired mental functions and altered behavior. Scientists are exploring whether heavy alcohol use, which occurs in a substantial number of HIV-infected people, may enhance the severity of HIV disease and its progression to AIDS. To date, the effects of alcohol on the immune function of brain cells and the susceptibility of those cells to HIV infection, as well as on the progression from HIV infection to AIDS have been ambiguous, writes Dr. Dieter J. Meyerhoff. Brain imaging studies have suggested, however, that both HIV infection and alcohol abuse have an effect on the brain’s energy metabolism and cellular metabolism, and that alcohol abuse augments the adverse effects of HIV on certain brain molecules. Dr. Meyerhoff also discusses how alcohol abuse may influence the effectiveness of treatment for HIV infection, either by interfering with the patient’s adherence to complex regimens or by reducing the effectiveness of the medications. (pp. 288–298)

## ALCOHOL USE AND THE RISK OF DEVELOPING ALZHEIMER’S DISEASE

Does alcohol use increase the risk for developing Alzheimer’s disease (AD)? Some of the detrimental effects of heavy alcohol use on the brain are similar to those observed with AD, and these similarities, along with the lack of standard diagnostic criteria for alcoholic dementia, make it difficult to study the relationship between alcohol use and AD. As Dr. Suzanne L. Tyas explains, similar biological mechanisms may be involved in the effects of AD and alcohol abuse on the brain. Epidemiologic studies have investigated the relationship between alcohol consumption and AD but have not provided strong evidence to suggest that alcohol use influences the risk of developing AD. Tobacco use may also affect the relationship between alcohol use and AD, and alcohol consumption may play a role in types of cognitive impairment other than AD. Further research is needed before the effect of alcohol use on AD is fully understood. (pp. 299–306)

